# Cooperative Binding of SRSF3 to Structured 3’ss-α Exon RNA during α Exon Inclusion in the ZO-1 mRNA

**DOI:** 10.3390/cimb45010039

**Published:** 2023-01-09

**Authors:** Tea Anastasia Ruiz-Luis, Carlos Ortuño-Pineda, José Manuel Galindo-Rosales, Odila Saucedo-Cárdenas, Jesús Valdés

**Affiliations:** 1 Departamento de Bioquímica, Centro de Investigación y de Estudios Avanzados del IPN, Mexico City 07360, Mexico; 2 Facultad de Ciencias Químico-Biológicas, Universidad Autónoma de Guerrero, Chilpancingo 39086, Mexico; 3 Departamento de Histología, Facultad de Medicina, Universidad Autónoma de Nuevo León, Monterrey 64460, Mexico

**Keywords:** ZO-1 isoforms, tight junction, alternative splicing, RNA–protein interaction, higher order RNA structure

## Abstract

ZO-1α+ and ZO-1α− proteins are expressed in hermetic and leaky tight junctions, respectively. Two cis-acting distant exonic elements partly activate the 240 nucleotide-long α exon producing the ZO-1α+ isoform. However, the elements within and around the α exon and their respective factors involved in its splicing are unknown. To study the dynamic interaction between SRSF3 and its bioinformatically predicted target sites around the 3’ss upstream of the α exon during its activation, we performed EMSA, crosslinking, and in vivo splicing assays by ZO-1 minigene expression and siRNA-mediated silencing in transfected cells. Using V1 RNase, we probed the possible formation of a hairpin RNA structure between the intronic and proximal exonic SRSF3 binding sites. The hairpin sufficed for complex formations in the EMSA. The interaction of SRSF3 with the intronic site promoted the cooperative binding of SRSF3 to the exonic site. Finally, SRSF3 restored α exon activation in SRSF3 knockdown transfectants. Altogether, our results show that SRSF3–hairpin RNA interaction is crucial in the early recognition of 3’ss for α exon activation. It remains to be explored whether SRSF3 recruits or stabilizes the binding of other factors or brings separate splice sites into proximity.

## 1. Introduction

Co-transcriptional alternative splicing of eukaryotic pre-mRNAs plays a major role in gene expression by selective intron removal generating a new shorter and mature mRNA formed by a diversity of combinatorial exons that are ultimately translated into protein, giving rise to huge amounts of different proteins involved in a variety of cellular mechanisms [1,2]. Early in spliceosomal assembly, cis-acting elements within the pre-mRNA must be recognized by trans-acting factors in order to regulate the inclusion/exclusion of alternative exons [3]. Major promoter and repressor factors correspond to SR (serine/arginine-rich) proteins and hnRNP proteins (heterogeneous nuclear ribonucleoproteins), which bind to exonic or intronic sequences, respectively, and reinforce spliceosomal splice site (ss) selection. Furthermore, both families of proteins have functional roles ranging from classical involvement in constitutive and alternative splicing to various post-splicing activities [4,5].

hnRNPs are modular proteins that bind to target pre-mRNAs through its RRM (RNA recognition motif) or KH (K homology) domains and influence the alternative splicing in different ways, including repressing spliceosomal assembly through multimerization on the exons, blocking the recruitment of snRNPs or by looping out entire exons [4]. SR proteins are widely characterized as a family of splicing regulators containing one or two RNA-binding (RBD or RRM) domains at their N-termini and a variable-length arginine/serine-rich (SR) domain at their C-termini. In most alternative splicing events, SR proteins mediate protein–protein or RNA–protein interactions, and coordinate interactions between two ss or ss and ESRs (exonic splicing regulators). The smallest member of this family, SRSF3, is known to regulate alternative splicing of several pre-mRNAs in a positive or negative manner [6] as occurs during CD44 and fibronectin pre-mRNA splicing, respectively [7,8].

In the search for the SRSF3 splicing-activation mechanism, in vitro approaches have shown that its RRM interacts with degenerated pyrimidine-rich sequences [9,10], whereas iCLIP (individual nucleotide resolution CLIP) was used to determine consensus binding sites in vivo [11], consistently finding CU-rich motifs as reported in vitro. Notably, the SRSF3 versatility in recognizing a variety of RNA sequences is probably due to the low specificity to contact all involved nucleotides by its RRM during SRSF3/RNA complex formation [9,12,13]. Finally, participation of SRSF3 in alternative splicing has an impact on many normal physiological processes, as well as in diseases, such as malignancy of several types of cancer [6,14] and dementia [15].

The alternative splicing of the 240 nucleotides (nt) long and regulated α exon of the ZO-1 pre-mRNA renders two tight junction (TJ) protein isoforms, ZO-1α+ and ZO-1α− [16], and the TJ permeability depends on the preferential expression of one of the two isoforms. Because the splice sites flanking the α exon are weak, its inclusion is controlled by two exonic splicing regulatory elements in the flanking constitutive exons [17]. In this study, we investigated the role of SRSF3 in the recognition of the 3’ss in front of the alternative α exon and analyzed the dynamics of SRSF3 interaction during α exon activation.

## 2. Materials and Methods

### 2.1. Cell Cultures and Protein Purification

MDCK and SHSY-5Y cells (ATCC Nos. CCL-34 and CRL-2266, respectively) were used to obtain protein extracts and for transfection experiments. Cells were cultured in a DMEM medium (Gibco-BRL) as described [17]. Nuclear extract (NEx) and SRSF proteins were purified as described [18,19]. SRSF3 was purified by elution from gel slices of SDS-PAGE fractionated SRSF extracts, and the purification procedure was monitored by Western blot.

### 2.2. Primers, Constructs, In Vitro Transcription, and RNase Probing

All the primer sequences used in this study are shown in Supplementary Figure S1. For the plasmid pp20 construct, the cDNA of the human SRSF3 was amplified by RT-PCR with primers SR20f and SR20r, and the product was sequenced and cloned in the pcDNA3 expression vector. The pcαAS minigene plasmid [17] was used as the template for site-directed mutagenesis using the respective primer pairs sM1-aM1 for 3’I-mut and sM2-aM2 for 3’e-mut. For the PCR amplification of the templates for in vitro transcription, the indicated primers sets were as follows: 3’wt (5αT7-44); 3’I-E (5αT7-Antisense-α); 3’I (5αT7-Anti αC); and 3’Δ (5αT7-44 and then cut with DraI). Meanwhile, the mutated plasmids pcαAS/3’I-mut and pcαAS/3’e-mut were templates for probes 3’I-mut and 3’e-mut (primers 5αT7-44). For template generation, PCR reactions were carried out with Platinum^®^ Taq DNA polymerase for 35 cycles at 95 °C for 30 s, 55 °C for 30 min, and 60 °C for 1 min. Synthetic transcripts were labeled with [α-^32^P]UTP as described [17] using the PCR products as substrates. For RNase probing, 3’ end-labeled synthetic transcripts were subjected to RNase A (20 fg/mL), RNase T1 (4–10 U/µL), or RNase V1 (4–10 U/µL) treatments, except that the RNAs were allowed to refold for 7 min, and were incubated in the presence or absence of 380 ng of purified SRp20 for 15 min at room temperature before RNase treatments [20].

### 2.3. Transfections and In Vivo Splicing Assays

**Figure 1:**
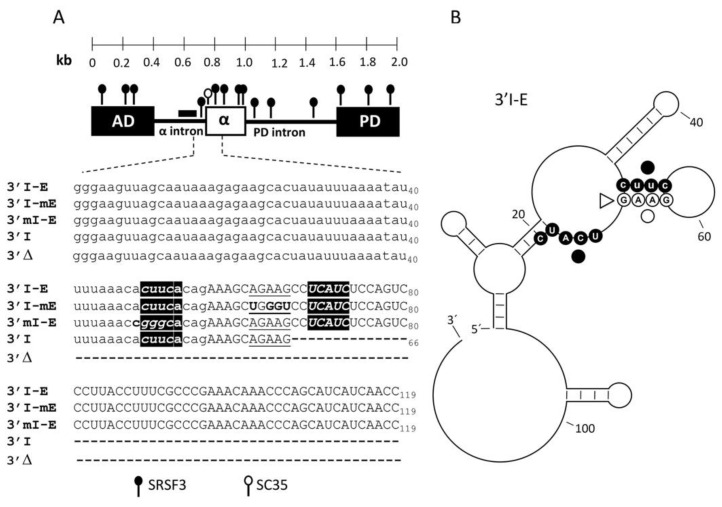
SRSF3 and the use of intron–exon boundaries are essential for the inclusion of alternative α exon in the ZO-1 mRNA. (**A**) Map of the α exon and the flanking introns and exons. The multiple SRSF3 (closed circles, boxed in black), SC35 (open circle, underlined), and hnRNP/Sam68/SLM-2 (black bar, uuuaaaa element) binding sites predicted in the depicted region are shown. The nucleotide sequences of the transcripts derived from the wild-type (3’ I-E), mutants (3’I-mE, and 3´mI-E), and deletion (3’I, and 3’Δ) minigenes used in this work are shown underneath. (**B**) Lowest energy secondary structure mFold model of the intron–exon boundaries in front of α exon. The SRSF and SC35 sites are marked with closed and open circles, respectively. Residue G66 is marked with an open arrowhead.

MDCK or SHSY-5Y cells were transfected with the indicated plasmids or siRNAs using Lipofectamine 2000 (Invitrogen, Waltham, MA, USA), and 24 to 48 h post-transfection, the total RNA of the cell cultures was purified (RNeasy mini kit, QIAGEN cat. 74104). The cDNAs for ZO-1 and actin were monitored by RT-PCR amplification using primers pairs (C3-B3, Act1-Act2, and BG1-BG2) as described in Figure 1A, and the amplification conditions were the same as those for in vitro transcription. SRSF3 knockdown experiments were performed in MDCK cells as previously described [8].

### 2.4. UV Crosslinking and EMSA

Proteins from MDCK NEx were UV-crosslinked for 20 min at a wavelength of 254 nm on ice to 250 fmol (~10^5^ cpm) of the labeled synthetic transcripts. Labeled proteins were resolved in 10% SDS PAGE. For the EMSA, splicing reactions were assembled in 10 µL of a binding buffer (50 mM Hepes-KOH, pH 7.6, 40 mM MgCl_2_, 20 mM spermidine, 0.1 mM DTT, 0.5 mM EDTA, 1.5 mM ATP and 20% Glycerol) containing 10 mg of nuclear extract and 0.4 mg/mL of tRNA, and were incubated for 15 min at room temperature. A total of 2000 cpm of ^32^P-labelled substrate RNA was added and incubated for 15 min at 24 °C. Samples were loaded on a 4.0% native polyacrylamide gel, electrophoresed, and visualized by autoradiography. In super-shift experiments, 1µg of antibodies was added before or after the corresponding probe; the non-related antibody was a mouse anti-IgG. For NEx SRSF3 depletion, NEx was incubated with 100 ng of the anti-SRSF3 monoclonal antibody, and after the addition of a 1:10 dilution of protein A-Sepharose (Zymed), samples were incubated overnight at 4 °C in agitation and centrifuged at 10,000 rpm for 3 min. SRSF3-depleted supernatants were recovered and used in EMSA experiments. A 1X molar ratio of homologous or heterologous probes was used to compete for the complex formation of the transcripts (10 fmol).

### 2.5. Western Blot

Nuclear protein extracts were then boiled in a Laemmli sample buffer (Cat. 1610737, Bio-Rad, Hercules, CA, USA), resolved by SDS-PAGE, and transferred to nitrocellulose membranes (Biorad, cat 1620112). The membranes were blocked for 2 h with 5% BSA. Specific bands were detected with primary antibodies and chemiluminescence using Clarity ECL Western blot substrates (Biorad, 1705060S, Hercules, CA, USA). Resolved bands were analyzed with the ImageJ software (version 1.5.3).

## 3. Results

### 3.1. The Exon α Architecture

In MDCK cells, the splicing of the alternative α exon is regulated by two exonic elements in the flanking constitutive exons in the ZO-1 pre-mRNA. The absence of consensus sequences in the 3’ss and in the branch point sequence, the lack of enhancer elements within the α exon, and the fact that substitutions of nearly the whole α exon do not improve its inclusion suggests that α exon recognition may be finely defined by trans-acting factors, most likely SRSF proteins whose binding sites might be localized in the upstream flanking intron [17]. In this study, using a minigene containing the α exon and its flanking introns and exons, we explored the context of sequences for α exon by in silico analysis. Particularly, we searched for splicing regulatory elements, as well as for the most probable secondary structure of the 3’ss in front of the α exon. The results showed a large number of binding sites for positive and negative splicing factors along the minigene, which included hnRNPs, Sam68, SLM-2, TIA-1, TIAL1, Nova 1, SRSF3, and SC35, among others (Figure 1A). The arrangement and redundancy of the hnRNP and SRSF3 in the intronic or exonic sequences, respectively, suggested a regulatory switch during ZO-1 mRNA processing, and SRSF3 probably has a key role in the mRNA recognition during the α exon inclusion.

Previous evidence suggested no essential splicing regulatory binding sites within the α exon [17]; therefore, we focused on the two SRSF3 binding sites surrounding the weak 3’ss in front of the α exon: the intronic site CUUCA at positions −8 to −4 from the 3’ss, whose structural analyses show that it could bind to the second site; and the exonic site CUCAUC at positions +12 to +17 in the α exon (Figure 1A), suggesting a possible SRSF3 interaction with an RNA hairpin structure (Figure 1B).

### 3.2. The 3’ss in Front of the α Exon Is Recognized by SRSF3 In Vitro

**Figure 2:**
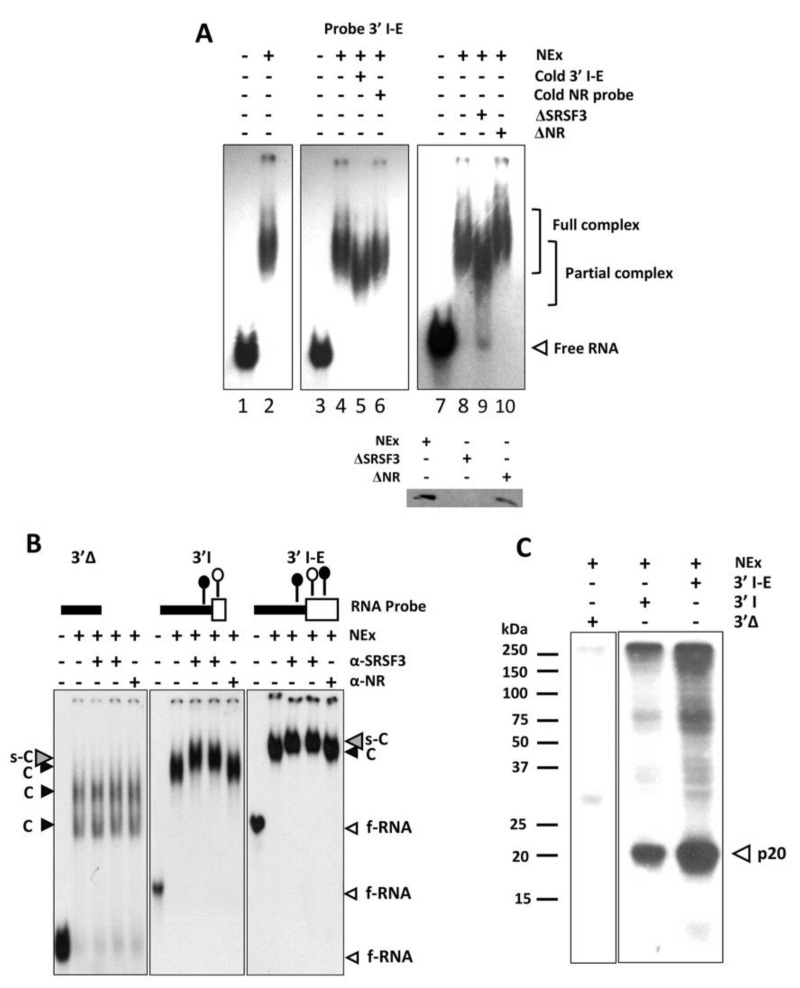
SRp20 directly recognizes the 3’ss in front of the α exon. (**A**) EMSA experiments were carried out with the 3’I-E transcripts and whole NEx. SRSF3/RNA interaction (lanes 2, 4, and 8) was challenged with the cold 3’I-E probe (lane 5) but not significantly with the unlabeled unrelated (cold NR) probe (lane 6). SRSF3-depleted nuclear extract (ΔSRSF3) failed to form the complex properly (lane 9), but depletion of NEx with unrelated antibodies (ΔNR) did not affect complex formation. Immunodepletions are shown in the inset. (**B**) Ten fmol of the different probes, plus 10 µg of MDCK NEx, and 800 ng of anti-SRSF3 or anti-non-related antibodies were used for super-shifts (s-C, gray arrowheads). Free probe (f-RNA, white arrowheads) and shifted complexes (C, black arrowheads) are also shown. (**C**) Direct interaction between SRSF3 and RNA was evidenced by crosslinking. The probe containing both binding sites showed a strong signal of a 20 kDa protein (p20, white arrowhead), which is less intense in the probes containing one or no SRSF3 binding sites.

To explore the interactions of SRSF3 around the 3’ss in front of α exon, different experimental approaches were conducted using several radiolabeled transcripts (Figure 1A). In the EMSA, the wild-type 3´I-E probe containing both SRSF3 binding sites around the intron–exon boundaries formed complexes with MDCK nuclear extracts (Figure 2A). Complex formation was compromised when either a cold 3’I-E probe or SRSF3-depleted MDCK NEx was added to the reaction but not with a cold unrelated probe or with unrelated antibodies (Figure 2A). The antibody anti-SRSF3 super-shifted the complexes formed between MDCK NEx and the probes 3’I-E and 3’I containing the intronic/exonic or the intronic SRSF3 binding sites, respectively. Interestingly, the intronic SRSF3 binding site alone was enough to shift the complex and no evident super-shift was observed with the probe 3’Δ lacking the intronic and exonic SRSF3 binding sites (Figure 2B). To further explore the direct interaction of SRSF3 with the transcripts, UV crosslinking experiments were carried out. In agreement with the super-shift experiments, a twenty kDa protein from the MDCK nuclear extract was crosslinked to the 3’I-E and 3’I probes but not to the 3’Δ (Figure 2C), although further evidence is needed to confirm the identity of the 20 kDa protein observed.

### 3.3. SRSF3 Cooperatively Binds around the 3’ss in a Structure-Dependent Way

**Figure 3:**
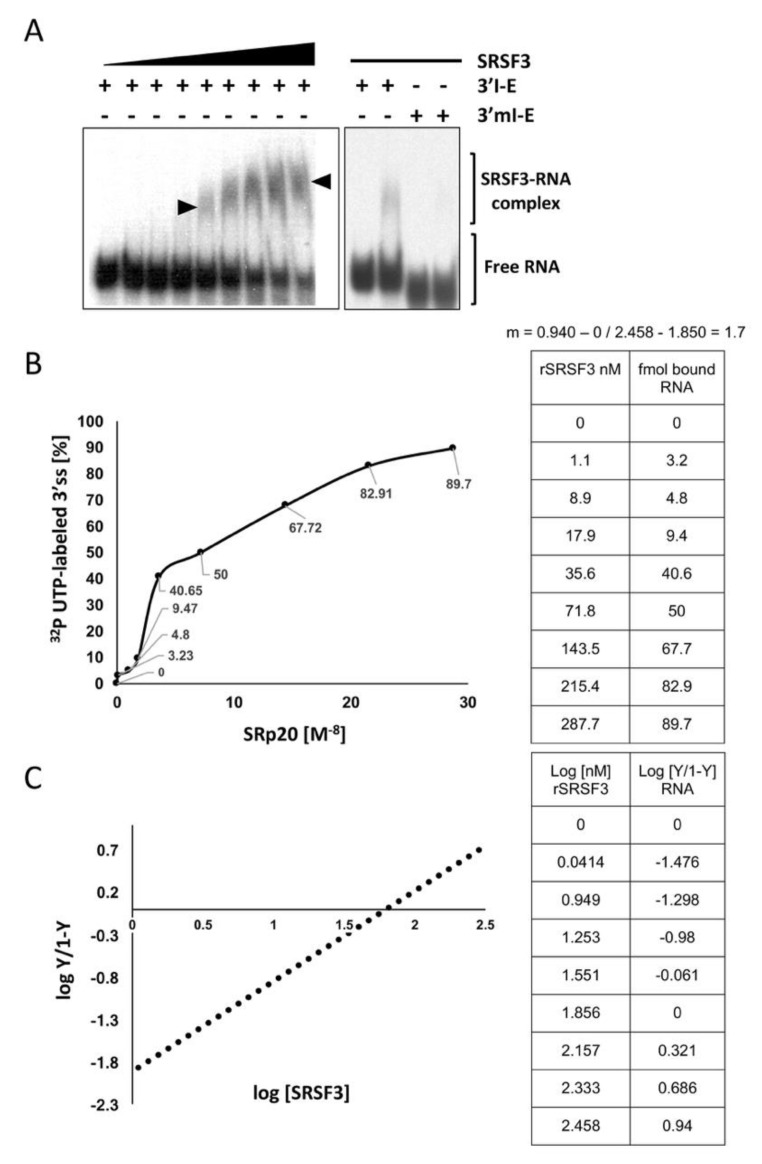
SRSF3 cooperatively binds in front of the 3’ss. (**A**) EMSA experiments using the probe containing two SRSF3 binding sites (3’I-E) and increasing concentrations of SRSF3 were added. No SRSF3 binding complexes were produced with the 3´mI-E probe. (**B**) The density of the complexes SRSF3/RNA and free probes were measured using ImageJ software and used for calculation. The plot shows the kinetics of the SRSF3/RNA interaction. (**C**) A Hill plot derived from the data (tables to the right) indicates that there is more than one active SRSF3 binding site (X-axis intersection > 1).

To study the SRSF3 interaction dynamic, we performed EMSA experiments using increasing concentrations of purified SRSF3 and the probe containing both SRSF3 binding sites. Figure 3A shows a discernible complex that migrates less into the gel (with no intermediate bands) as the concentration of the recombinant SRSF3 increases. As previously described [21], such a migration pattern is indicative of cooperative interactions between two SRSF3 molecules and the SRSF3 binding sites. The sigmoid kinetics of the SRSF3/RNA interaction (Figure 3A,B) and the Hill plot intersect and slope (m = 1.7) (Figure 3C) are also suggestive of cooperative binding between the recombinant and the intronic and exonic SRSF3 target sites.

**Figure 4:**
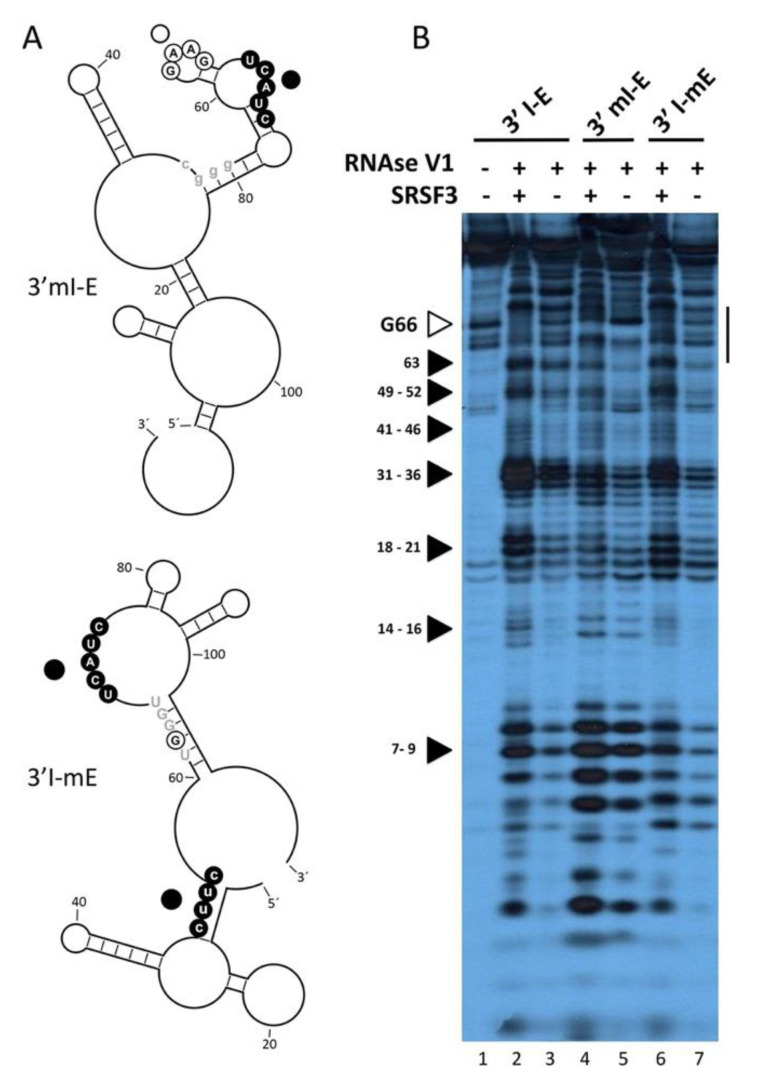
SRSF3—intronic binding imposes structural changes. (**A**) Lowest energy models of the intronic (3’mI-E) and structural (3’I-mE) mutants. The sequences of the α intron and exon appear in lower- and upper-case lettering, respectively. Residues 55–57 correspond to the 3’ss intron/exon boundary. (**B**) 3´end-labeled 3’I-E and 3’I-mE synthetic RNAs were incubated with RNase V1 (10^−4^ U/µL), with (+) or without (−) 380 ng of purified SRSF3. The digested products were resolved by 20% denaturing electrophoresis. The bar to the right indicates the SRSF3 footprint; residues G63–G66 are indicated by arrowheads; in agreement with the predicted lowest energy secondary structure, black arrowheads at positions 49–52, 41–46, 31–36, 18–21, 14–16, and 7–9 show the hypersensitive sites resulting from SRSF3–RNA interaction.

Interestingly, the probe 3’mI-E contains a mutation in the intronic SRSF3 binding site but not in the exonic site, failing to interact with SRSF3 protein, and reinforcing the previous idea that the intronic site is necessary for the appropriate interaction of the exonic SRSF3 binding site. To explore whether SRSF3 imposes changes in the 3´ss hairpin-loop secondary structure predicted by Mfold RNA tools [22], RNase-probing experiments were carried out. These experiments have the added benefits of delineating the interaction site of SRSF3 and confirming the predicted structure of the 3’ss (Figure 4A). Both free RNA and RNA–SRSF3 mixtures were treated with RNase V1 (which cleaves double-stranded RNA in a non-sequence-specific manner). A comparison of the various digestion products of the probes 3’I-E, 3’mI-E (mutant in the intronic site CUUC→CGGG), and 3’I-mE (mutant in an exonic non-related site AGAAG→AGGAG) identified residues involved in the secondary structure and those in contact with the protein. As expected, the presence of SRSF3 decreased the intensity of several RNase V1 bands (between residues 62–67) in the wild-type 3´I-E probe (Figure 4B, lanes 1–3), but not in the intronic mutant 3’mI-E (lanes 4–5), suggesting that SRSF3 binding to the intronic site stabilized double-stranded RNA interactions, probably exposing other nearby single-stranded sites. To delineate the relevance of the loop structure, we analyzed the structural mutant 3’I-mE that did not disturb the SRSF3 binding sites but altered the hairpin arrangement of such sequences. Interestingly, the effect was similar to that observed for intronic mutants (Figure 4B, lines 6–7), suggesting a structural dependence for SRSF3 interaction.

### 3.4. SRSF3 Regulates the Splicing of the α Exon In Vivo via Its Intronic Binding Site

**Figure 5:**
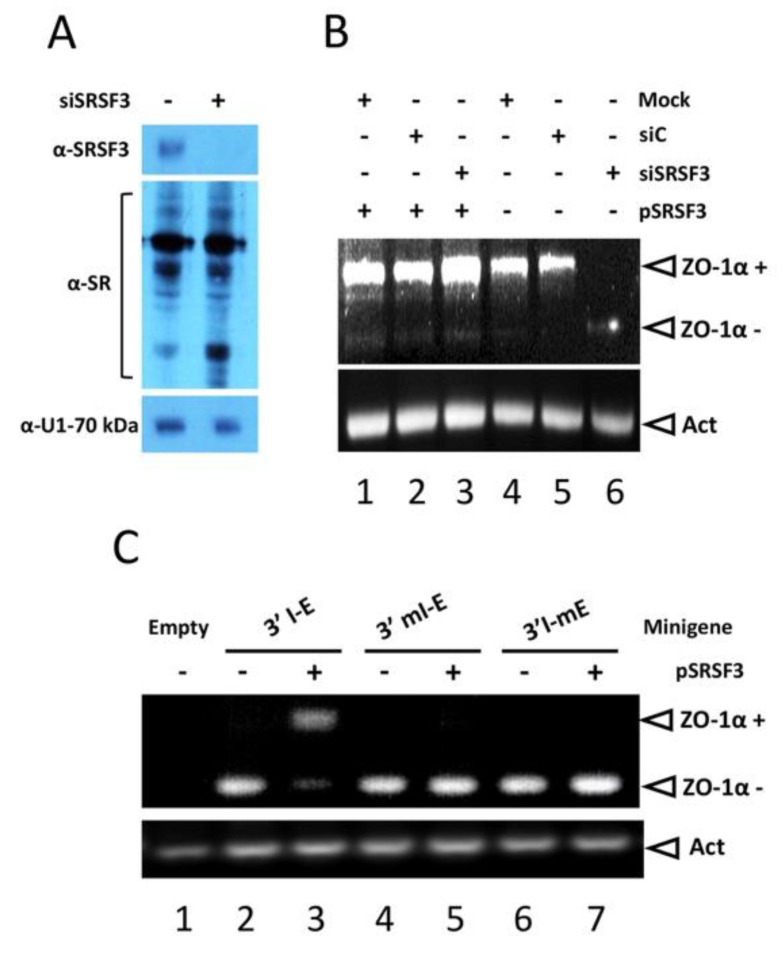
SRSF3 activates the α exon inclusion via its intronic binding site. MDCK cell cultures were mock-transfected or transfected with siRNAs against SRSF3 (siR-SRSF3) or non-related siRNAs (siR-Control). SRSF3 knockdown was monitored by Western blot with anti-SRSF3 antibody and compared to the pan-SR antibody and anti-U1-70 kDa loading control (**A**). Then, the expression of the ZO-1α+ (914 nt) and ZO-1α− (674 nt) mRNA variants were analyzed by RT-PCR in duplicate transfectants where the SRSF3 cDNA (pSRSF3) was complemented (**B**). Finally, SHSY-5Y cells were transfected with pcDNA3 empty vector, pcαAS/3’I-E minigene, or with pcαAS minigene bearing mutations in the intronic SRSF3 binding site (pcαAS/3’I-mut) or the structural nonrelated sequence (pcαAS/3’e-mut) with (+) or without (−) pSRSF3 construct co-transfection (**C**). The expression of ZO-1α+ and ZO-1α− mRNA variants was analyzed by RT-PCR.

To address the role of SRSF3 in the splicing of the α exon, SRSF3 was knocked down, and the expression of the ZO-1 mRNA variants was monitored. siRNA-mediated SRSF3 knockdown was specific and did not disturb the expression of other SRSF or spliceosomal proteins, e.g., U1-70 kDa (Figure 5A). In contrast with the treatment with the control siRNA, siRNA against SRSF3 inhibited the α exon activation, which was restored in the pSRSF3 co-transfection experiments (Figure 5B, compare lanes 3 and 6), clearly indicating that SRSF3 is essential for α exon inclusion. To demonstrate the importance of the intronic SRSF3 binding site, the intronic mutant 3’mI-E and the exonic mutant 3’I-mE were generated by site-directed mutagenesis in the pcαAS minigene construct. We used SHSY-5Y cells (which are unable to produce both ZO-1 isoforms, Figure 5C, lane 1) and performed in vivo splicing assays transfecting the wild-type pcαAS and the mutant pcαAS minigenes, either alone or in the presence of the SRSF3 cDNA (pSRSF3). The transfection of the 3’I-E pcαAS minigene rendered only the ZO-1α− mRNA variant (Figure 5C, lane 2), as previously reported [17], whereas the ZO-1α+ variant was obtained when SRSF3 was over-expressed (lane 3). However, when we transfected the intronic mutant 3’mI-E pcαAS plasmids with or without SRSF3 co-transfection, the SHSY-5Y cells were not able to produce the ZO-1α+ isoform, suggesting a role of the intronic site in α exon inclusion (lanes 4–5). Notably, mutant 3’I-mE rendered only the ZO-1α− variant (lanes 6–7), emphasizing the higher-order structure requirement for 3’ss recognition in vivo.

## 4. Discussion

Extensive information on alternative splicing mechanisms has been gathered in the past 20 years, including a collection of spliceosomal proteins and non-snRNP splicing factors involved [1]. Among others, the SR proteins are critical alternative splicing regulators in different systems [23], although some details on the splicing factors of intron recognition remain unknown. Furthermore, the role of SRSF3 in a huge number of processes and pre-mRNAs has been studied. Here, we report how SRSF3 interaction on the 3’ss triggers the inclusion of the alternative α exon in the ZO-1 mRNA.

In agreement with the fact that SRSF3 is essential for mouse embryo development, activating several alternative exons of proteins involved in the TJ biogenesis, including ZO-1 [7,8,14,24,25,26,27], we found additional pieces of evidence supporting the notion that SRSF3 binding to the 3’ss in front of the α exon is the limiting step for its inclusion.

The computational analysis showed an intricate arrangement of SRSF3 binding sites on the α exon and its flanking introns and exons. Notably, two SRSF3 binding sites surrounding the weak 3’ss in front of the α exon were localized. The exonic SRSF3 binding (AAAGCAGAAG) is similar to the motifs involved in the activation of the CD44 variable exon v9 (ACAUGAAGGCUUGGAAGAAGAUAAAGAC) [7] and in nuclear export (ACAACAAGAAGAGCGCCAUCAU) [28], whereas the intronic SRSF3 binding (CUUCA) coincided with the 9G8 and SRSF3 binding sites reported previously [9,10,12,13,29]. Using an in vitro approach, we were able to elucidate the dynamic SRSF3/RNA interaction and, in vivo, we demonstrated the need for SRSF3 to activate α exon inclusion in the ZO-1 mRNA in transfected MDCK cells.

Exon activation by SRSF3 binding sites in exonic enhancers has been reported previously [29], and the influence of 3’ss structure on pre-mRNA splicing has also been documented. On the one hand, the RNA secondary structure sequesters cis-acting elements, thus selecting alternative 3´ss or distal BP [30,31,32]. On the other hand, SRSF [33,34] and hnRNP proteins are able to bind structured RNA and might compete with other factors of the splicing machinery [35]. Nevertheless, to our knowledge, this is the first report to show the stabilizing role of SRSF3 on the 3’ss secondary RNA interactions with a positive effect on the selection of a 3´ss, resulting in the inclusion of a whole exon cassette. Because SRSF3 stabilizes the secondary structure of the 3’ss, it probably contributes to U2AF2/U2AF1 recruitment, either by providing a splicing-enhancer-like function or by contorting the RNA, bringing separate splice sites into proximity [36], thus promoting splicing, which deserves detailed studies.

In conclusion, here we showed the cooperative recognition of two SRSF3 molecules of a higher-order structure formed by an intronic and exonic SRSF3 binding sites around a 3’ss, collaborating together to activate an alternative exon.

## Data Availability

Not applicable.

## Supplementary Materials

The following supporting information can be downloaded at: https://www.mdpi.com/article/10.3390/cimb45010039/s1, Figure S1: Oligonucleotides and its use in this study.
