# Supplementary material for: Cooperative Binding of SRSF3 to Structured 3’ss-α Exon RNA during α Exon Inclusion in the ZO-1 mRNA
Source: Curr Issues Mol Biol. 2023 Jan 9;45(1):593–603. doi: 10.3390/cimb45010039 (PMC9857539; doi:10.3390/cimb45010039)
Supplement: Supplementary file 1 [file cimb-45-00039-s001.zip › cimb-2068209-Figure S1.pdf]

Supplementary Figure S1. Oligonucleotides and its use in this study.

USE	Primer name/sequence 5'-3'
Transcription	44-(GTTGATGATGCTGGGTTTG)
	5αT7(TAATACGACTCACTATAGGGGAAGTTAGCAATAAAGAGAAGC)
	Antisense α (CTTCTGCTTTCTGTGAAGTG)
	Anti-αC (TCTGCTTTCTGTGAAGTG)
RT PCR	C3 (ATGAAGACACAGATACAGAAGGCGGG)
	B3 (TCATAGCGTGGTCTGCTGTCATAGGAC)
	Act1 (TCACGCACGATTTCCCTCTCAG)
	Act2 (ATTTGGCACCACACTTTCTACA)
	BG1 (CAACTTCATCCACGTTCACC)
	BG2 (GAAGAGCCAAGGACAGGTAC)
Directed mutagenesis	sM1 (TTTTAAACCGGGCACAGAAAGCAGAAGCCTCATCTCCAG)
	aM1 (CTGGAGAT GAGGCTTCTGCTTTCTGTGCCCGGTTTAAAA)
	sM2 (CACTTCACAGAAAGCTGGGTCCTCAT CTCCAG)
	aM2 (CTGGAGATGAGGACCCAGCTTTCTGTGAAGTG)
Cloning	SR20f (ATGCATCGTGATTCCTGCC)
	SR20r (AAGGACATGTGGTGAAAACC)
